# Morning pulmonary artery pressure measurements by CardioMEMS are most stable and recommended for pressure trends monitoring

**DOI:** 10.1007/s12471-021-01590-7

**Published:** 2021-06-10

**Authors:** S. Crnko, J. J. Brugts, J. F. Veenis, N. de Jonge, J. P. G. Sluijter, M. I. F. Oerlemans, L. W. van Laake

**Affiliations:** 1 Department of Cardiology and Experimental Cardiology, University Medical Center Utrecht, Utrecht, The Netherlands; 2 Regenerative Medicine Centre, Circulatory Health Laboratory, University Medical Center Utrecht, Utrecht, The Netherlands; 3 Department of Cardiology, Erasmus MC University Medical Center, Rotterdam, The Netherlands; 4 Utrecht University, Utrecht, The Netherlands

**Keywords:** Heart failure, CardioMEMS, Circadian rhythm, Individualised therapy, Telemonitoring

## Abstract

**Aims:**

The CardioMEMS HF system is used to measure pulmonary artery (PA) pressures of patients with heart failure (HF). The goal of this study was to determine the impact of time in the daily PA pressure measurements, considering variance and influence of circadian rhythms on cardiovascular pathophysiology.

**Methods and results:**

The study included 10 patients with HF with reduced ejection fraction (LVEF < 40%; New York Heart Association class III). Individual daily PA pressures were obtained by CardioMEMS sensors, per protocol, measured up to six times throughout the day, for a period of 5 days. Differences between variation of morning versus evening PA pressures were compared with Wilcoxon signed-rank test.

Mean PA pressures (mPAP) increased from a morning value of 19.1 ± 2 mm Hg (8 am; mean ± standard error of the mean [SEM]) to 21.3 ± 2 mm Hg late in the evening (11 pm; mean ± SEM). Over the course of 5 days, evening mPAP exhibited a significantly higher median coefficient of variation than morning mPAP (14.9 (interquartile range [IQR] 7.6–21.0) and 7.0 (IQR 5.0–12.8) respectively; *p* = 0.01). The same daily pattern of pressure variability was observed in diastolic (*p* = 0.01) and systolic (*p* = 0.04) pressures, with diastolic pressures being more variable than systolic at all time points.

**Conclusions:**

Morning PA pressure measurements yield more stable values for observing PA trends. Patients should thus be advised to consistently perform their daily PA pressure measurements early in the morning. This will improve reliability and interpretation of the CardioMEMS management, indicating true alterations in the patient’s health status, rather than time-of-day-dependent variations.

**Supplementary Information:**

The online version of this article (10.1007/s12471-021-01590-7) contains supplementary material, which is available to authorized users.

## What’s new?

- Pulmonary artery pressures are most stable in the mornings
- Highest pulmonary artery pressures are observed late in the evening
- Daily pulmonary artery pressures are advised to be measured early in the morning
- Standardised CardioMEMS measurements will improve reliability of the pressure values

## Introduction

Heart failure (HF) has become a major socio-economic burden as it is characterised by episodes with acute decompensation, requiring hospitalisation, and is associated with increased morbidity and mortality [1, 2]. The CardioMEMS system, an implantable wireless pulmonary artery (PA) pressure monitor, was shown to reduce hospitalisations by improving HF management in the CHAMPION trial [3]. Since the 2016 European Society of Cardiology guidelines on HF [4], ambulatory haemodynamic monitoring may be considered (IIb indication) in symptomatic patients with previous HF hospitalisation. Subsequent analyses and data obtained in the clinical setting showed an improved quality of life and reduced morbidity and mortality using ambulatory haemodynamic monitoring [5–8].

Currently, HF patients with a CardioMEMS sensor are instructed by the manufacturer to measure PA pressures in the morning. However, it has not yet been investigated whether morning indeed yields the most precise PA pressure measurements, while some patients may prefer a different time for practical reasons, or may vary the time of their measurement considerably. Additionally, while daily patterns of PA pressures measured in a consistent manner are currently not available, CardioMEMS provides the opportunity to study them. Previous studies on PA pressures in HF suggest the existence of diurnal rhythms [9, 10]. Given the emerging importance of these circadian rhythms in cardiovascular pathophysiology [11], we aimed to define the most optimal time of day for measuring PA pressures in HF patients.

## Methods

### Study design

A total of 10 consecutive patients (New York Heart Association [NYHA] class III), who had received a CardioMEMS sensor according to the current guidelines [4], participated in this study. The study was conducted at the University Medical Center Utrecht and Erasmus University Medical Center in accordance with the declaration of Helsinki and the Medical Research Involving Human Subjects Act. All participants provided written informed consent. Individual chronotypes were self-assessed using the Dutch morningness-eveningness questionnaire (VOA) and PA pressure measurements were extracted from the online system (merlin.net).

### Measurements of PA pressures

The CardioMEMS sensor was used to obtain individual daily measurements of PA pressures at six time points throughout the day (8 am, 11 am, 2 pm, 5 pm, 8 pm, 11 pm) for a period of two consecutive days, followed by three time points (8 am, 5 pm, 11 pm) for the next three consecutive days. Measurements were downloaded for analysis using the CardioMEMS online system (merlin.net).

### Statistical analysis

The coefficient of variation (CV) was used as a measure of relative variability of mean (mPAP), systolic (sPAP), and diastolic (dPAP) PA pressures, either within-day or between-day. The CV was calculated as the ratio of the standard deviation to the mean, and expressed as a percentage. Wilcoxon signed-rank test was used to compare differences between CVs of PA pressures, and Student’s t‑test was used to compare differences between morning and evening heart rate. A *p*-value of < 0.05 was considered statistically significant. All analyses were performed in IBM SPSS Statistics (v. 23).

## Results

### Patient characteristics

A total of 10 patients (mean age 59 ± 9 years, 60% male) were enrolled in this study. Patient characteristics are summarised in Tab. 1. All patients were in NYHA class III at the time of CardioMEMS implantation with diuretics and optimal guideline-directed medical therapy.

**Table 1:** Patient characteristics at the time of CardioMEMS implantation

		All subjects (*N* = 10)
Characteristics	Age (years)	59.0 ± 9
	Male sex	60.0
	BMI (kg/m^2^)	26.5 ± 3
	eGFR (ml/min/1.73 m^2^)	52.9 ± 16
	Heart rate (bpm)	69.9 ± 12
	Systolic blood pressure (mm Hg)	98.5 ± 12
	Diastolic blood pressure (mm Hg)	59.6 ± 9
	Mean arterial pressure (mm Hg)	72.6 ± 8
Laboratory assessment	NT-proBNP (pmol/l)	232.2 ± 164
	Sodium (mmol/l)	138.0 ± 2
	Potassium (mmol/l)	4.3 ± 0
	AST (U/l)	34.9 ± 21
	ALT (U/l)	33.2 ± 19
	LD (U/l)	266.9 ± 124
	Gamma-GT (U/l)	44.7 ± 46
	Alkaline phosphatase (U/l)	94.3 ± 45
	Hb (mmol/l)	7.9 ± 1
Comorbidities	Diabetes mellitus	30.0
	Myocardial infarction	50.0
	Atrial flutter/fibrillation	20.0
	Hypertension	10.0
	COPD	0.0
	OSAS	0.0
Cause of cardiomyopathy	iCMP	50.0
	DCM	50.0
Severity of heart failure	Ejection fraction (%)	23.9 ± 4
	NYHA III (%)	100.0
	HTx	10.0
Medication	Beta blocker	70.0
	RAASi (ARNi)	100.0 (50.0)
	MRA	100.0
	Diuretics	100.0

Values are mean ± standard deviation or percentage

*ALT* alanine aminotransferase, *ARNi* angiotensin receptor-neprilysin inhibitor, *AST* aspartate aminotransferase, *BMI* body mass index, *COPD* chronic obstructive pulmonary disease, *DCM* dilated cardiomyopathy, *eGFR* estimated glomerular filtration rate calculated with the Chronic Kidney Disease Epidemiology Collaboration equation [13], *Gamma-GT* gamma-glutamyl transferase, *Hb* haemoglobin, *HTx* heart transplantation, *iCMP* ischaemic cardiomyopathy, *LD* lactate dehydrogenase, *LVEF* left ventricular ejection fraction, *MRA* mineralocorticoid receptor antagonist, *NT-pro-BNP* N-terminal pro-brain natriuretic peptide,* NYHA class* New York Heart Association functional classification of heart failure severity [4], *OSAS* obstructive sleep apnoea syndrome, *RAASi* renin-angiotensin-aldosterone system inhibitor

### Diurnal variation of pulmonary artery pressure

**Fig. 1:**
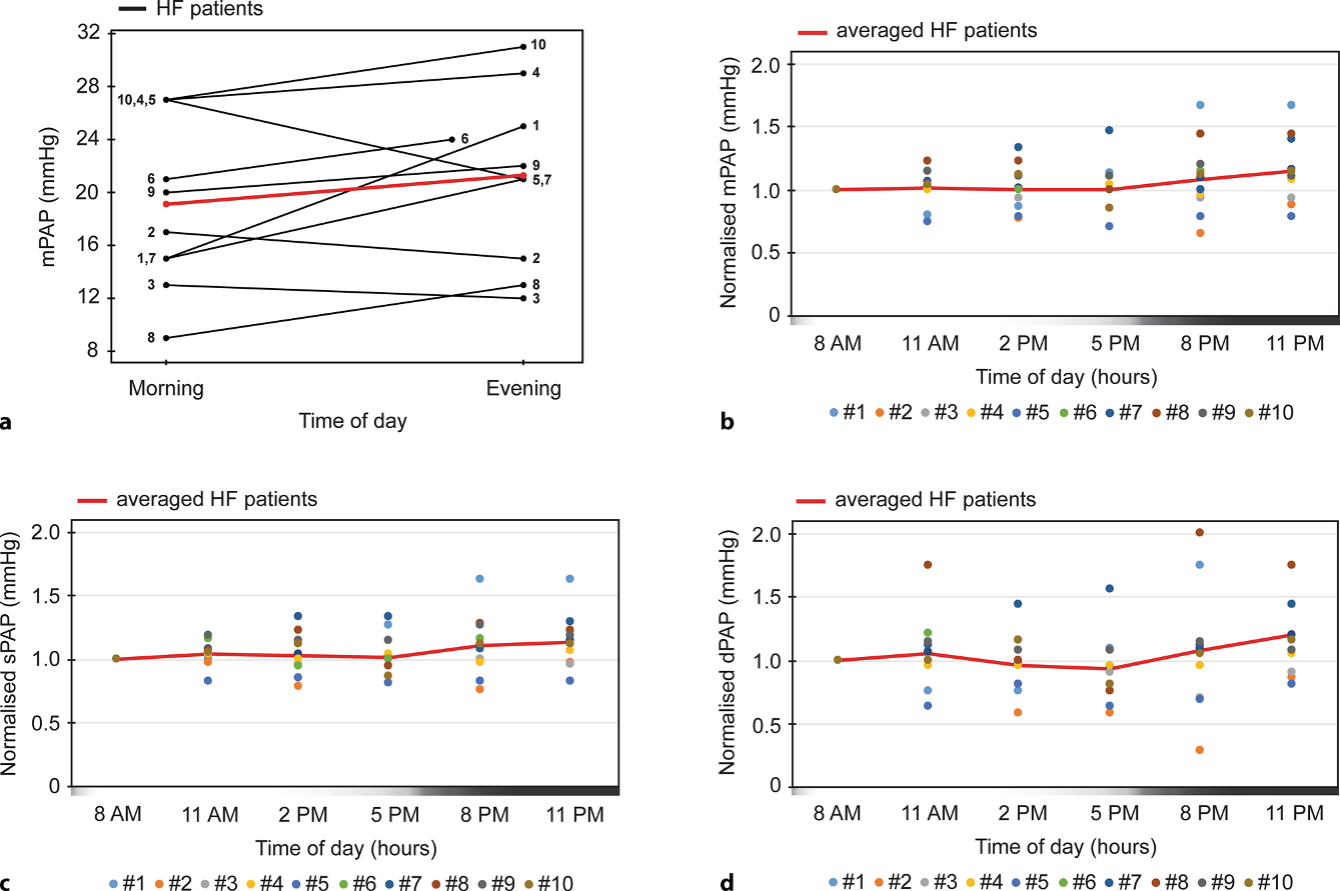
Pulmonary artery pressure increases in heart failure patients in the evening. **a** Morning (8 am) and evening (11 pm for all patients; except for patient 6, 8 pm) mPAP (mm Hg) within-day distribution of 10 individual HF patients. Mean values of morning and evening measurements each are indicated with red line. **b**, **c**, **d** Within-day distribution of normalised PA pressures (mm Hg) in each HF patient (*N* = 6/patients; except for patients 3 and 6, *N* = 5). Per patient, each measurement was normalised against the value of the first time point of either (**b**) mPAP, (**c**) sPAP or (**d**) dPAP. Red line represents averaged normalised values of all patients per time point (*N* = 10; except for time points 11 am and 11 pm, *N* = 9). Gradient bar depicts time of day (white = day, black = night). *dPAP* diastolic pulmonary artery pressure, *HF* heart failure, *mPAP* mean pulmonary artery pressure, *PAP* pulmonary artery pressure, *sPAP* systolic pulmonary artery pressure

Within a 24‑h period, in 7 out of 10 patients mPAP values increased from morning to evening. The mean mPAP value was 19.1 ± 2 mm Hg in the morning (8 am; mean ± SEM) and 21.3 ± 2 mm Hg in the evening (11 pm; mean ± SEM) (Fig. 1a), thus a relative increase of 11.5% (range: −6 mm Hg = −28.6% to +10 mm Hg = +66.7%). The biggest difference was noted in patient 1, with a morning mPAP of 15 mm Hg which increased to 25 mm Hg at 11 pm.

Next, in order to visualise the overall diurnal patterns of patients with different absolute values, normalised mPAP, sPAP and dPAP were analysed (*N* = 10, Fig. 1b–d). Again, the same pattern of highest PA pressures was observed late in the evening.

### Pulmonary artery pressure is more stable in the mornings than in the evenings

**Fig. 2:**
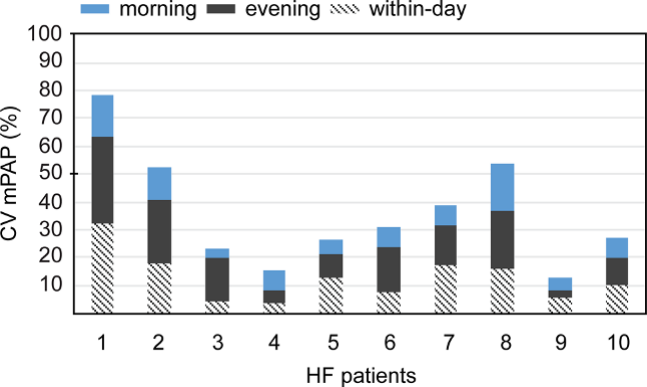
Pulmonary artery pressure in heart failure patients varies less when measured in the mornings than in the evenings. Values are calculated based on measurements of 5 consecutive days, either in the morning (8 am; *N* = 5 for each patient) or in the evening (11 pm; *N* = 5/patient; except for patient 6, *N* = 2), or within a 24‑h period (*N* = 6/patient; except for patients 3 and 6, *N* = 5). Each bar represents a single HF patient. CV (%) is calculated as the ratio of the standard deviation to the mean. *CV* coefficient of variation, *HF* heart failure, *mPAP* mean pulmonary artery pressure

Since clear reproducible differences between morning and evening measurements were observed, we investigated whether it is relevant to strictly prescribe morning versus evening PA pressures in daily practice using the between-day CV. Interestingly, the between-day CV of mPAP, sPAP and dPAP was significantly higher in the evening than in the morning measurements (*p* = 0.01, *p* = 0.04, and *p* = 0.01 respectively; Tab. 2). When considering mPAP, the median CV of evening pressures was twice as high compared with morning (14.9 (interquartile range [IQR] 7.6–21.0) and 7.0 (IQR 5.0–12.8) respectively) (Tab. 3). Additionally, the within-day variation was higher than the between-day CV for morning mPAP during 5 consecutive days, illustrating the importance and stability of daily morning measurements (Fig. 2). sPAP and dPAP followed the same trend (Supplementary Table S1), with dPAP being less stable than mPAP or sPAP both in mornings and evenings (Tab. 2). Although patients’ activity could influence the PA pressures, it does not seem to be the cause of the observed high variation in the evening values, based on the CV of heart rate measurements (6.1 (IQR 4.5–12.0) in the morning and 3.2 (IQR 2.6–9.2) in the evening, *p* = 0.17; Supplementary Table S2). Intriguingly, patients who exhibited the highest PA pressure variation in the evenings scored as either moderate morning or pronounced morning chronotypes on the VOA questionnaire (data not shown), pointing to the influence of individual diurnal rhythms.

**Table 2:** Degree of variation between morning and evening PA pressure measurements

	CV (%) of mPAP (IQR)	CV (%) of sPAP (IQR)	CV (%) of dPAP (IQR)
Morning	7.0 (5.0–12.8)	7.0 (5.3–10.7)	9.4 (6.6–21.2)
Evening	14.9 (7.6–21.0)	11.4 (6.1–17.1)	15.6 (10.3–31.7)
*P* value	0.01	0.04	0.01

Median CV values are calculated based on measurements of 5 consecutive days, either in the morning (8 am; *N* = 5/patient) or in the evening (11 pm; *N* = 5/per patient; except for patient 6 *N* = 2). CV (%) is calculated as the ratio of the standard deviation to the mean. Wilcoxon signed-rank test was used to test differences between morning and evening CV of mPAP, sPAP and dPAP

*CV* coefficient of variation, *dPAP* diastolic pulmonary artery pressure, *IQR* interquartile range, *mPAP* mean pulmonary artery pressure, *sPAP* systolic pulmonary artery pressure

**Table 3:** Between-day variation of mPAP (mm Hg) in heart failure patients

Patient number	Time of day	Minimum mPAP (mm Hg)	Maximum mPAP (mm Hg)	Mean mPAP (mm Hg)	CV (%)
1	Morning	15.0	22.0	17.6	15.4
	Evening	12.0	25.0	17.4	30.6
2	Morning	14.0	19.0	16.2	11.9
	Evening	10.0	18.0	13.6	22.4
3	Morning	13.0	14.0	13.2	3.4
	Evening	11.0	16.0	12.6	15.5
4	Morning	24.0	29.0	26.6	6.8
	Evening	26.0	29.0	27.2	4.8
5	Morning	24.0	27.0	25.6	5.2
	Evening	17.0	21.0	19.6	8.5
6	Morning	19.0	23.0	20.8	7.1
	Evening	19.0	24.0	21.5	16.4
7	Morning	15.0	18.0	16.6	6.9
	Evening	18.0	26.0	22.6	14.2
8	Morning	9.0	14.0	12.2	16.8
	Evening	11.0	19.0	14.8	20.5
9	Morning	20.0	22.0	20.6	4.3
	Evening	21.0	22.0	21.4	2.6
10	Morning	26.0	31.0	28.6	7.3
	Evening	25.0	32.0	29.8	9.3

Values are calculated based on measurements of five consecutive days, either in the morning (8 am; *N* = 5/patient) or in the evening (11 pm; *N* = 5/patient; except for patient 6 *N* = 2). CV (%) is calculated as the ratio of the standard deviation to the mean

*CV* coefficient of variation, *mPAP* mean pulmonary artery pressure

## Discussion

In this study, we provided more scientific evidence supporting the current CardioMEMS recommendations to measure PA pressures in the morning. The present data demonstrate the stability of morning PA pressures, suggesting that any observed differences in morning values are more likely reflecting clinically relevant changes in heart failure status, rather than merely natural diurnal variation. Changes in evening PA pressures could be attributed to circadian fluctuations and apparently correct themselves overnight, at least in clinically stable patients.

While diurnal properties of PA pressures were previously explored [9, 10], this is the first study to directly assess its clinical relevance in an ambulatory setting. We sought to determine the most optimal time of day for PA pressure measurements based on intra- and inter-day variation. In order to do so, we opted for a translational set-up by omitting time points during the night (between 12 am and 6 am), when maximal PA pressures were previously noted [9].

In most of the subjects, PA pressure increased in the evening. The evening rise of PA pressure is in line with other studies [9, 10], and ascribed to its diurnal nature. Furthermore, over the course of 5 days, PA pressure exhibited different degrees of time-of-day-dependent variation. Morning measurements were consistently stable in the vast majority of patients, while noticeable fluctuations were usually observed in the evening. Diastolic PA pressure varied more than systolic PA pressure, regardless of the time of day. Pronounced variation in the evenings could be explained by patients’ daily activities, e.g. food and fluid daily intake variation, effect of different medications, as well as physical activity. All of these factors influence important physiological processes directly and via sympathetic nerve system or renin-angiotensin-aldosterone system (RAAS) activation, and can cause PA pressures to fluctuate [12].

Collectively, our data offers a 2-fold evidence to support the current CardioMEMS recommendations to measure PA pressures in the morning. Firstly, we show that PA pressures exhibit a diurnal rhythm, with lowest pressures measured in the morning, and reaching their peak in the evening. Therefore, by measuring PA pressures consistently in the mornings, any changes in pressures could be ascribed to a change in the patient’s condition. For example, if the pressure would first be measured in the morning (= lowest PA pressure), with a follow-up measurement in the evening (= highest PA pressure), it could indicate a deterioration of the patient’s condition, while it actually only reflects a mere circadian fluctuation. If the pressure is always measured in the morning, any deviating recording of PA pressures would be a clear indication that an intervention is needed. Secondly, as previously mentioned, PA pressures may be affected by patient’s activity during the day, including exercise, and food, fluid, and medication intake. The susceptibility of PA pressures to these daily changes could explain why evening PA pressures were least stable in the majority of subjects during a course of five days. Combined with their diurnal properties, highest and least consistent PA pressures are observed in the evening. Thus, morning PA pressure measurements are more reliable and better reflect the true changes in the patient’s condition (e.g. worsening of heart failure), rather than showing the influence of daily activities (e.g. food intake, exercise, etc.) which will reset until the next day. In conclusion, morning PA pressure measurements, as opposed to evening measurements that are prone to external influences, will give a clear indication for intervention if relevant increase in values is observed.

## Limitations

Our study included a relatively low number of patients (*N* = 10), however, this was sufficient to show consistent changes in daily PA pressures. A large set of time points per patient was provided, both within the 24 h and during the course of five days. This gives a better representation of the individual PA pressure fluctuations, substantiating derived conclusions about morning PA pressure stability.

## Conclusion

**Fig. 3:**
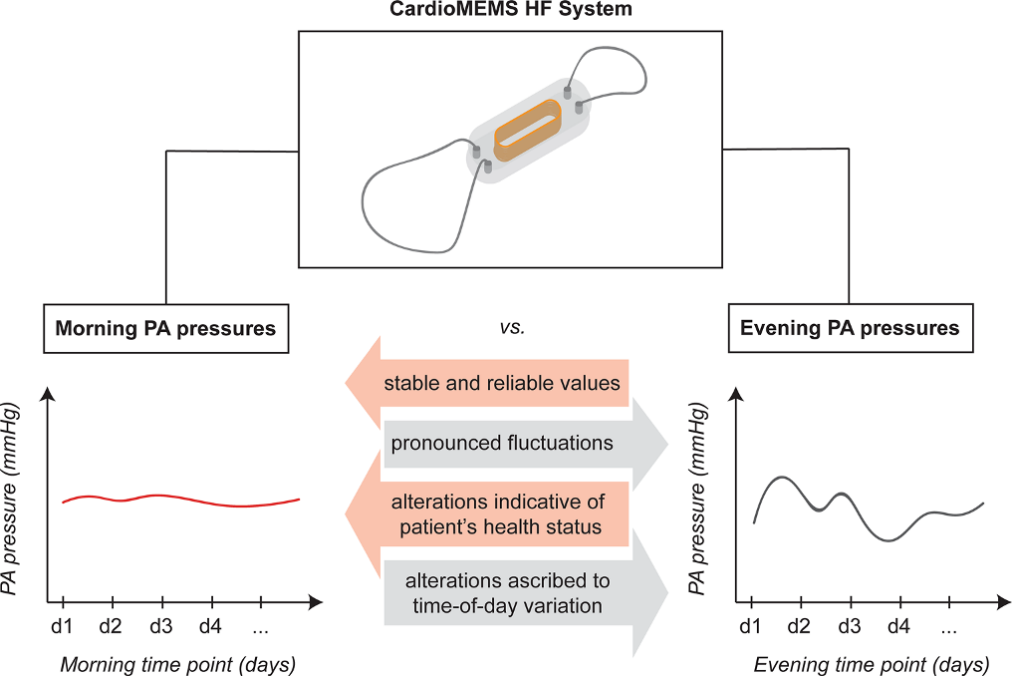
Morning pulmonary artery pressure measurements provided by the CardioMEMS sensor, as opposed to evening measurements, yield more stable and reliable values. *HF* heart failure, *PA* pulmonary artery

Given the pronounced variation in ambulatory PA pressures during the day, standardised morning PA pressure measurements will improve reliability and interpretation of the values provided by the CardioMEMS sensor (see Take-home Fig. 3). Clinicians will be able to ascertain which changes of the PA pressures indicate true alterations in the patient’s health status (e.g. worsening of HF) and which merely reflect natural diurnal properties.

## Supplementary Information

Table overview of between-days variation of sPAP, dPAP, and heart rate in heart failure patients.
