# Supplementary material for: Morning pulmonary artery pressure measurements by CardioMEMS are most stable and recommended for pressure trends monitoring
Source: Neth Heart J. 2021 Jun 10;29(7-8):409–14. doi: 10.1007/s12471-021-01590-7 (PMC8271080; doi:10.1007/s12471-021-01590-7)
Supplement: Supplementary file 1 — Table overview of between-days variation of sPAP, dPAP, and heart rate in heart failure patients. [file 12471_2021_1590_MOESM1_ESM.docx]

**Morning pulmonary artery pressure measurements by CardioMEMS are most stable and recommended for pressure trends monitoring**

**Short title: The importance of morning CardioMEMS measurements**

S. Crnko^1,2*^ and J. J. Brugts^3*^, J. F. Veenis^3^, N. de Jonge^1^, J. P.G. Sluijter^1,2,4^, M. I.F. Oerlemans^1#^ and L. W. van Laake^1,2#^

^1^ Department of Cardiology, Experimental Cardiology Laboratory, University Medical Centre Utrecht, Utrecht, the Netherlands

^2^ Regenerative Medicine Centre, Circulatory Health Laboratory, University Medical Centre Utrecht, Utrecht, the Netherlands

^3^ Department of Cardiology, Erasmus MC University Medical Center, Rotterdam, the Netherlands

^4^ Utrecht University, Utrecht, the Netherlands

^*^ These authors share first authorship and contributed equally.

^#^ These authors contributed equally.

**Correspondence:**

L.W. van Laake

Department of Cardiology

Division of Heart and Lungs

University Medical Centre Utrecht

Utrecht, the Netherlands

L.W.vanLaake@umcutrecht.nl

**Supplementary data**

**Supplementary Table S1 Between-day variation of sPAP and dPAP in heart failure patients**

| **Patient number** | **Time of day** | **Minimum** | | **Maximum** | | | **Mean** | | **CV (%)** | |
| --- | --- | --- | --- | --- | --- | --- | --- | --- | --- | --- |
|  |  | **sPAP**  **(mmHg)** | **dPAP**  **(mmHg)** | **sPAP**  **(mmHg)** | | **dPAP**  **(mmHg)** | **sPAP**  **(mmHg)** | **dPAP**  **(mmHg)** | **sPAP**  **(mmHg)** | **dPAP**  **(mmHg)** |
| *1* | Morning | 19.0 | 12.0 | 27.0 | 19.0 | | 23.4 | 14.2 | 13.0 | 19.5 |
|  | Evening | 19.0 | 8.0 | 31.0 | 21.0 | | 24.0 | 13.6 | 20.2 | 39.1 |
| *2* | Morning | 27.0 | 5.0 | 35.0 | 9.0 | | 30.6 | 6.4 | 11.5 | 26.1 |
|  | Evening | 23.0 | 2.0 | 33.0 | 8.0 | | 28.0 | 5.0 | 14.3 | 44.7 |
| *3* | Morning | 21.0 | 9.0 | 23.0 | 10.0 | | 22.0 | 9.4 | 4.5 | 5.8 |
|  | Evening | 19.0 | 8.0 | 26.0 | 11.0 | | 21.4 | 8.8 | 12.6 | 14.8 |
| *4* | Morning | 30.0 | 20.0 | 37.0 | 24.0 | | 33.6 | 22.4 | 7.5 | 6.8 |
|  | Evening | 32.0 | 21.0 | 36.0 | 24.0 | | 33.8 | 22.6 | 4.4 | 5.9 |
| *5* | Morning | 34.0 | 14.0 | 40.0 | 18.0 | | 37.2 | 15.8 | 6.4 | 9.4 |
|  | Evening | 28.0 | 9.0 | 33.0 | 14.0 | | 31.2 | 12.0 | 6.6 | 15.6 |
| *6* | Morning | 27.0 | 13.0 | 34.0 | 16.0 | | 31.4 | 13.8 | 8.6 | 9.4 |
|  | Evening | 27.0 | 12.0 | 36.0 | 16.0 | | 31.5 | 14.0 | 20.2 | 20.2 |
| *7* | Morning | 27.0 | 9.0 | 31.0 | 12.0 | | 29.0 | 10.4 | 5.5 | 12.9 |
|  | Evening | 33.0 | 12.0 | 42.0 | 17.0 | | 37.8 | 15.0 | 10.1 | 15.6 |
| *8* | Morning | 18.0 | 4.0 | 23.0 | 9.0 | | 20.8 | 6.8 | 10.4 | 26.3 |
|  | Evening | 21.0 | 5.0 | 31.0 | 11.0 | | 25.2 | 8.6 | 16.0 | 29.2 |
| *9* | Morning | 27.0 | 14.0 | 30.0 | 16.0 | | 28.6 | 14.8 | 4.0 | 5.7 |
|  | Evening | 30.0 | 14.0 | 32.0 | 15.0 | | 31.0 | 14.8 | 3.2 | 3.0 |
| *10* | Morning | 44.0 | 19.0 | 51.0 | 23.0 | | 47.6 | 21.0 | 6.2 | 7.5 |
|  | Evening | 42.0 | 17.0 | 52.0 | 23.0 | | 48.8 | 21.4 | 8.5 | 11.7 |

Values are calculated based on measurements of 5 consecutive days, either in the morning (8 am; *N*=5/patient) or in the evening (11 pm; *N*=5/patient; except for patient 6, *N*=2). CV (%) is calculated as the ratio of the standard deviation to the mean.

*CV* coefficient of variation, *dPAP* diastolic pulmonary artery pressure, *mPAP* mean pulmonary artery pressure, *sPAP* systolic pulmonary artery pressure

**Supplementary Table S2 Between-day variation of heart rate in heart failure patients**

| **Patient number** | **Time of day** | **Maximum** | **Minimum** | **Mean** | **CV (%)** | ***P* value** |
| --- | --- | --- | --- | --- | --- | --- |
|  |  | **HR**  **(bpm)** | **HR**  **(bpm)** | **HR**  **(bpm)** | **HR**  **(bpm)** | **Morning vs. evening HR** |
| *1* | Morning | 105.0 | 89.0 | 97.6 | 7.0 | 0.85 |
|  | Evening | 103.0 | 95.0 | 98.2 | 3.5 |  |
| *2* | Morning | 65.0 | 57.0 | 61.4 | 4.7 | 0.26 |
|  | Evening | 63.0 | 53.0 | 58.4 | 7.1 |  |
| *3* | Morning | 65.0 | 60.0 | 62.8 | 3.1 | 0.03 |
|  | Evening | 69.0 | 65.0 | 66.8 | 2.7 |  |
| *4* | Morning | 102.0 | 76.0 | 85.8 | 11.7 | 0.84 |
|  | Evening | 90.0 | 79.0 | 84.4 | 5.3 |  |
| *5* | Morning | 88.0 | 77.0 | 81.0 | 5.2 | 0.00 |
|  | Evening | 69.0 | 65.0 | 67.0 | 2.4 |  |
| *6* | Morning | 94.0 | 71.0 | 76.6 | 12.8 | 0.73 |
|  | Evening | 89.0 | 86.0 | 87.5 | 2.4 |  |
| *7* | Morning | 61.0 | 56.0 | 58.0 | 4.0 | 0.49 |
|  | Evening | 76.0 | 54.0 | 60.6 | 15.5 |  |
| *8* | Morning | 86.0 | 66.0 | 75.6 | 9.4 | 0.40 |
|  | Evening | 81.0 | 75.0 | 78.4 | 2.9 |  |
| *9* | Morning | 88.0 | 79.0 | 81.8 | 4.7 | 0.54 |
|  | Evening | 83.0 | 78.0 | 80.2 | 2.7 |  |
| *10* | Morning | 65.0 | 44.0 | 49.4 | 17.8 | 0.21 |
|  | Evening | 67.0 | 43.0 | 51.6 | 17.6 |  |

Values are calculated based on measurements of 5 consecutive days, either in the morning (8 am; *N*=5/patient) or in the evening (11 pm; *N*=5/patient; except for patient 6, *N*=2). CV (%) is calculated as the ratio of the standard deviation to the mean. *P* values indicate differences between morning and evening HR values per patient, calculated with Student’s t-test.

*CV* coefficient of variation *HR* heart rate
